# Unilateral Absence of Mental Foramen with Surgical Exploration in a Living Human Subject

**DOI:** 10.1155/2016/1971925

**Published:** 2016-02-24

**Authors:** Murat Ulu, Elif Tarim Ertas, Fatih Gunhan, Meral Yircali Atici, Huseyin Akcay

**Affiliations:** ^1^Department of Oral and Maxillofacial Surgery, Faculty of Dentistry, Izmir Katip Celebi University, 35640 Izmir, Turkey; ^2^Department of Oral and Maxillofacial Radiology, Faculty of Dentistry, Izmir Katip Celebi University, 35640 Izmir, Turkey

## Abstract

The mental foramen (MF) is an important anatomic landmark of the mandible, in which the somatic afferent sensory nerve of the mandibular nerve emerges as mental nerve and blood vessels. The identification and actual location of MF are important in order to avoid sensory dysfunction or paresthesia due to mental nerve injury. In the literature there are some rare reports on the anatomical variations of the MF such as its location or presence of accessory foramina. The present report describes the absence of mental foramina on the left side of the mandible, as detected by cone-beam computed tomography before impacted tooth removal and observed directly during surgery.

## 1. Introduction

The mental foramen (MF) nerves divide into several branches to provide sensorial innervation of the angle of the mouth with its angular branch: the skin of the lower lip, oral mucosa, and gingiva up to the second premolar with its medial and lateral inferior labial branches and the skin of the mental region with its mental branch [1]. MF is generally located in the area of premolar region as bilateral oval or round openings, although race and ethnicity may affect its location [2]. Radiographically, the MF can be seen as a round or oval radiolucent area on both the right and left sides of the mandible [3, 4].

The identification and actual location of MF are important in order to avoid sensory dysfunction or paresthesia during any surgical procedures in this area, as well as achieving effective anesthesia [3, 5, 6]. Since the conventional radiographs such as periapical and panoramic films provide two-dimensional images of the mandible [2, 7], the MF may not be observed in some cases due to the superimposition of anatomic landmarks [5, 8], the pattern of trabecular bone [4], and the thinning of the mandible [5]. In the literature, variations of the MF such as its location or presence of accessory foramina are reported in several articles but the absence of MF is extremely rare [5, 5].

We present here a case of unilateral absence of the mental foramen in a living patient detected on cone-beam computed tomography (CBCT) images and surgical exploration during lower impacted 2nd and 3rd molar extraction. According to our knowledge there is a very extremely rare case report in the literature which indicates absence of mental foramen with CBCT image and by surgical exploration.

## 2. Case Report

**Figure 1:**
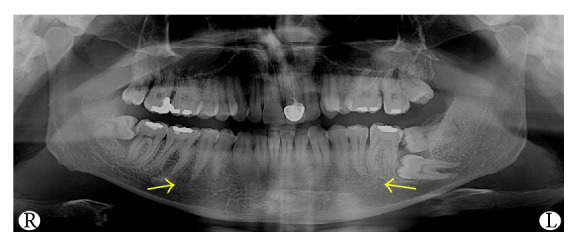
20-year-old patient's panoramic radiograph in which the images of the right MF and the end of the left mandibular canal are pointed by the arrows.

**Figure 2:**
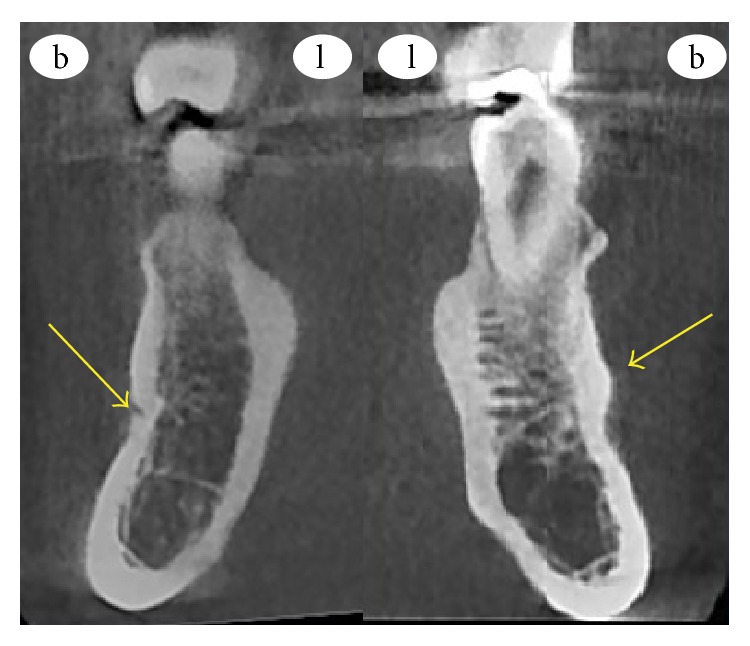
Coronal CBCT slice in which the openings of the right MF and the left protuberance at the MF area are pointed by the arrows.

**Figure 3:**
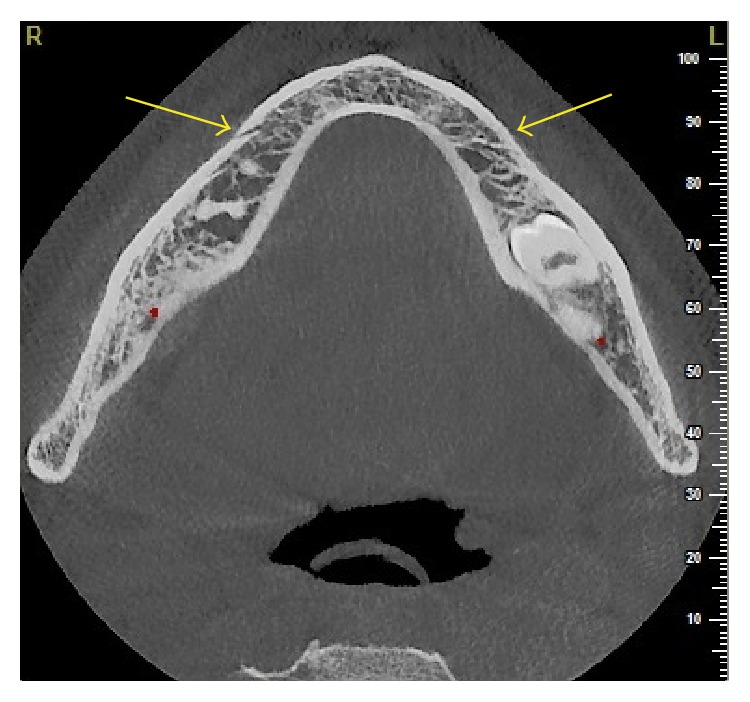
Transverse CBCT slice of the right MF is pointed by the arrow and the left MF area protuberance is pointed by the arrow.

**Figure 4:**
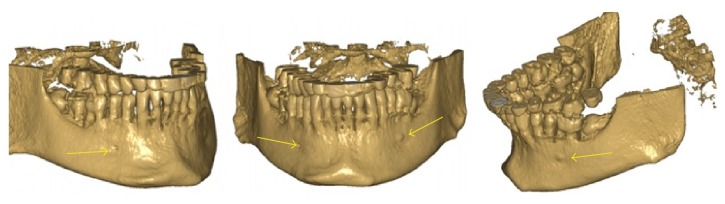
3D reconstruction of the patient's right side, left side, and frontal appearance showing hypoplastic foramen and protuberance at the MF area.

**Figure 5:**
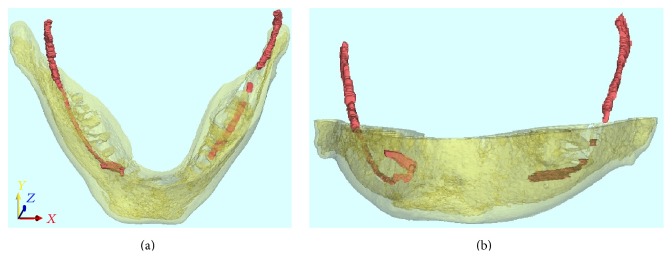
Segmentation of the mandibular canal by using CBCT images with Mimics software.

A 20-year-old male patient was referred to Izmir Katip Celebi University Faculty of Dentistry with a chief complaint of pain in left posterior mandible. A panoramic radiograph disclosed the presence of fully impacted mandibular 2nd and 3rd left molars with horizontally overlapped position (Figure 1). A radiolucent area around impacted molars and bone resorption distal side of 1st molar was observed. Extraction was decided for both of the impacted teeth. In order to evaluate the relation between the impacted 2nd molar and inferior alveolar nerve, CBCT (NewTom 5G®, QR, Verona, Italy) examination was performed before surgery. All axial, coronal, and 3D images were carefully explored. During CBCT examination an uncommon anatomic variation drew our attention. In the left mandible MF could not be seen and on the right side there was a slight foramen between the 1st and 2nd premolars. We assessed axial and coronal images (Figures 2 and 3) carefully and then made a 3D construction (Figure 4). On the left side MF was absent but the patient did not complain about sensory deficiency. Using the fine slices from the CBCT, we analysed the mandibular images with Mimics software (Materialise, Leuven, Belgium) and reconstructed the data into 3D images. By segmenting the mandibular canals, we revealed their courses within the mandible. Although right mandibular nerve exists into the MF at the 1st premolar region, the left canal could not be identified after the region of tooth 35 (Figure 5). We could not identify incisive canal on the left side.

**Figure 6:**
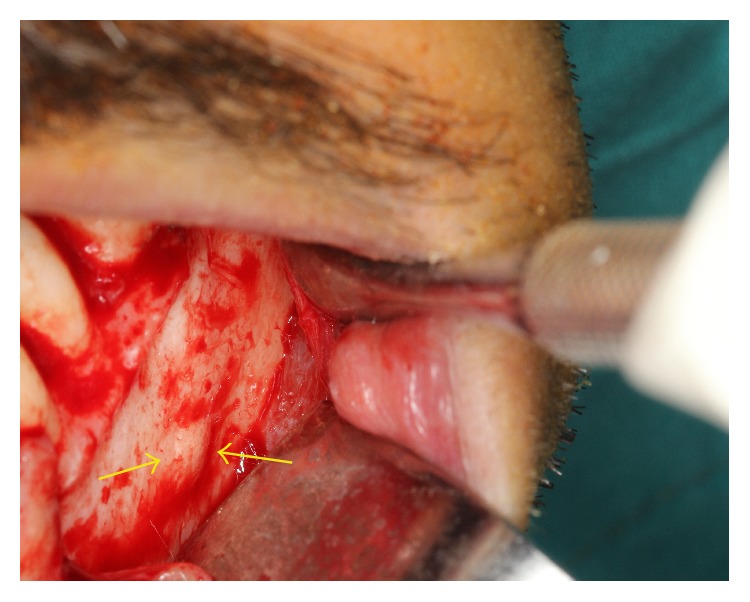
Clinical appearance of the protuberance at the mental foramen area on the left mandible.

Prior to surgical removal of the impacted teeth the patient was informed about paresthesia. Inferior alveolar nerve block anesthesia was achieved with 4 mL local anesthetic (Ultracaine D-S Fort, Sanofi Aventis, Istanbul, Turkey). Sulcular incision, which extended to the middle of the 1st premolar, was combined with two releasing incisions and then a mucoperiosteal envelope flap was reflected. At the 1/3 apical level of the 2nd premolar, bone prominence was seen; there was no foramen (Figure 6). After providing a clear exposure the 3rd molar was divided into small pieces with tungsten round and fissure bur and then extracted with a Bein elevator. After that 2nd molar was extracted with the same procedure. Finally, the granulation tissue was scraped from the cavity and the flap was repositioned in its original place and then sutured with 3-0 silk (Dogsan, Trabzon, Turkey). Antibiotic and nonsteroidal anti-inflammatory drugs were prescribed after surgery. We did not notice nerve disturbance during suture removing seance.

## 3. Discussion

In recent years with the improvement of implant dentistry and its growing popularity, the possibility of surgical procedures near the MF has increased. Therefore, detailed anatomical knowledge of the region and MF's variations is crucial. MF is an important anatomical landmark of the mandible during surgical procedures in order to achieve effective mental nerve block anesthesia and to prevent mental nerve injuries of the lower jaw [9].

The location of MF shows changes in different races [2]. It is usually located apical to the second premolar or between apices of the premolars and first molars [9]. In the first years of life before tooth eruption, the MF exists closer to the alveolar margin; in time it is observed closer to the inferior border, and with aging related to tooth loss and bone resorption the MF is found closer to the alveolar border [7].

Usually the MFs are observed as bilateral oval or round opening on the lateral surface of the mandible, but some variations of MF have been reported in the literature. However, accessory foramina, retromolar foramen, and abnormal courses of the inferior alveolar neurovascular bundle were recorded as normal variations in the studies [6, 10]. The size of MF varies individually. Greenstein and Tarnow [5] assessed the size of MF by using morphometric skull and reported the following mean values: height (3.47 mm; range 2.5–5.5 mm), width (3.59 mm; range 2–5.5 mm), and diameter (3.5 and 5 mm wide). In our case, we measured the dimensions of the right MF using transversal CBCT slices and values are as follows: height 1.1 mm, width 1.2 mm. The left MF of the patient was absent.

There is a limited publication about the exact prevalence of MF agenesis in the literature. De Freitas et al. [11] examined 1,435 dry human mandibles and they could not detect MF only in three cases. The limitations of 2D radiographs may be the possible explanation for this lack of data, because conventional panoramic radiographs are not reliable for true diagnosis due to the superimposition of teeth, trabecular pattern of bone, thinning of mandible, or patient positioning and processing errors. This imaging method provided insufficient information to diagnose accurately the width and height of the bone and exact relationship with the adjacent structures especially before implant surgeries. CBCT is a more effective method that provides three-dimensional (3D) imaging and exact linear measurements for presurgical assessment, evaluation of the bone quality, and the possible anatomic variations [2]. In addition, acquisition of 3D reconstructions and use of lower radiation doses in comparison to medical CT are some of other advantages of the system [1, 6] which may play a key role in elucidating details of these normal variations [9].

There is a rare case report about absence of MF in the literature. In 2011, da Silva Ramos Fernandes et al. [1] detected unilateral absence of the MF and hypoplasia on the other side in a patient undergoing CBCT prior to orthodontic examination which was the first case in the literature of MF absence seen in CBCT images of a living patient. In 2013 Matsumoto et al. [9] reported bilateral absence of the MF, which was detected incidentally by CBCT performed prior to dental implant surgery. In most recent case reported by Lauhr et al. [2], they incidentally discovered bilateral absence of MF during CBCT examination before implant surgery. In the present case report, unilateral MF agenesis which was detected incidentally by CBCT prior to impacted teeth extraction is represented in the literature.

The reason for the absence of the MF is unclear [9]. The underlying cause seems most likely to be congenital agenesis because in all of the reported cases (including our case) no evidence of trauma existed and the mandibles were otherwise healthy with normal morphology [7]. da Silva Ramos Fernandes et al. [1] detected unilateral MF absence in female patient's mandible and then they examined her mother CBCT; there was unilateral hypoplasia of the MF; they concluded that variations of the MF may partly arise through genetic factors.

Our patient did not complain of any nerve or developmental disturbances around his mentum and lower lip. In the previously mentioned case reports none of the authors reported any innervation or vascularization disturbance [1, 9]. Therefore the authors concluded that the mental nerve and blood vessels serving the mental region might be very thin but surely present and may run with an alternative course, which could go undetected [1, 9].

Particular attention should be paid to such variations as indicated in the literature since knowledge about the morphology of the jaw and position of the mandibular foramen helps dental and oral and maxillofacial surgeons to carry out successful local anesthetic blocks and surgical procedures without postoperative complications.
